# Physically Based Bubble Growth Model for Micro-Scale Foam Injection Moulding CFD Simulation

**DOI:** 10.3390/polym18202574

**Published:** 2026-10-19

**Authors:** Daniel C. Fritsche, Malte Schön, Christian Hopmann

**Affiliations:** 1 Chair for Plastics Processing Rheinisch-Westfälische Technische Hochschule Aachen University, Seffenter Weg 201, 52074 Aachen, Germany; 2 3M Deutschland GmbH, Carl-Schurz-Straße 1, 41453 Neuss, Germany

**Keywords:** foam injection molding (FIM), bubble growth, mass source, microcellular foaming, OpenFOAM, volume-of-fluid (VoF), multiscale simulation, bubble deformation, coalescence

## Abstract

Predicting spatially varying cell morphology in foam injection molding is required for microstructure-based part design but remains limited by spherical bubble-growth models. This study extends a compressible, viscoelastic volume-of-fluid framework with a diffusion-controlled bubble-growth model. Bubble-specific mass-transfer rates are calculated from geometry, temperature-dependent gas properties, interfacial concentration, and prescribed bulk gas concentration, and introduced iteratively as local mass sources in OpenFOAM. The model was evaluated for two polycarbonate–nitrogen parameter sets and two plate positions against experimental cross-sections. The approach produced continuous and monotonic bubble growth. The simulations reproduced through-thickness trends, including larger cells in the core, decreasing cell size toward the walls, and preferential deformation in the shear layers. The simulated trends in cell density and area agreed qualitatively with the experiments, and aspect ratios of 1.5–2.0 for one parameter set were comparable to measurements. Absolute morphology descriptors were not reproduced quantitatively because of the two-dimensional representation, simplified nucleation history, prescribed bulk gas concentration, and one-way coupling. The deformation results rely on a case-specific reduction to 10% of the macro-scale pressure difference. The model provides a physically motivated methodological framework for resolving bubble growth and qualitative spatial trends in foam morphology.

## 1. Introduction

### 1.1. Motivation and Microstructure Relevance

The accurate prediction of material behavior during the early design phase of plastic parts is increasingly important for reducing development costs, shortening development cycles, and minimizing experimental effort [1]. Virtual product development enables the evaluation of geometries, materials, and process conditions prior to manufacturing, thereby supporting more efficient and sustainable part design [1]. In this context, integrated computational approaches aim to link product engineering and manufacturing-process simulation, since the final performance of plastic parts is strongly influenced by their processing history [1].

For injection-molded parts, process conditions such as flow, cooling, and pressure evolution induce locally varying material states, including molecular orientation, residual stresses, crystallinity, and—in the case of foams—cellular morphology [2,3]. These local variations directly affect the mechanical response and dimensional stability of the final part, so that homogeneous material descriptions are often insufficient for accurate structural prediction [2,3,4]. Consequently, modern simulation strategies increasingly follow a process–structure–property–performance concept, in which process-induced inhomogeneities are transferred to subsequent material models [1,4].

This is particularly relevant for FIM, where the effective material behavior is governed by the local cellular structure. Foam injection-molded parts exhibit a heterogeneous skin–core morphology with compact outer layers and a cellular core, and their properties depend on local quantities such as cell size, cell density, and cell shape [5,6,7]. These morphological characteristics are not constant but evolve as a function of local pressure, temperature, and gas concentration during the process.

In this work, the term bubble refers to a gas domain surrounded by mobile plastic that melts during nucleation, growth, deformation, and coalescence. After solidification, the corresponding cavity in the foamed plastics is referred to as a cell. The term foam morphology denotes the collective properties of the solidified cellular structure, including cell density, size, shape, aspect ratio, orientation, and spatial distribution.

Experimental and numerical studies show that foam morphology is sensitive to processing conditions. For instance, changes in cavity pressure and pressure-drop rate strongly influence nucleation and resulting cell density [8,9], while melt and mold temperatures as well as gas dosage affect cell size and overall foam structure [10,11]. In addition, the type of blowing agent (e.g., carbon dioxide or nitrogen) influences solubility and diffusion behavior, leading to different cell morphologies [12,13,14]. During subsequent growth, cells can deform due to flow and expansion, often resulting in anisotropic or elongated structures in the core region [15,16,17], while coalescence between neighboring bubbles further modifies the morphology.

These findings highlight that foam morphology is inherently heterogeneous and process-dependent, making its accurate prediction a prerequisite for reliable material and component design.

### 1.2. Challenges of Modeling the Microstructure

Despite the well-understood physical mechanisms, the quantitative prediction of foam morphology remains challenging. Commercial and macro-scale injection molding simulations typically describe the foaming process using averaged quantities such as cell radius, cell density, or density distribution [18,19]. However, these parameters do not capture essential features such as cell shape, orientation, coalescence, or local gradients across the part thickness.

This limitation is critical because the properties of cellular materials depend not only on relative density but also on cell topology and morphology [15,20]. Thus, simulations based solely on averaged foam characteristics are often insufficient to predict local mechanical properties, especially for anisotropic or strongly heterogeneous structures. Although incorporating foam morphology into material models improves prediction quality, deviations in failure behavior and structural response remain [4].

Furthermore, macro-scale simulations are sensitive to model assumptions, calibration parameters, and numerical settings. Studies have shown that results depend strongly on the selected bubble-growth models and simulation setup, and that consistent prediction trends are not always achieved across different approaches [10,21].

A fundamental challenge is that foam morphology evolves through coupled processes—nucleation, growth, deformation, and coalescence—under transient thermomechanical conditions. In current simulations, these mechanisms are typically simplified, for example by assuming spherical bubbles and neglecting interactions such as deformation or coalescence [22,23,24,25,26]. As a result, existing models can provide useful, averaged information but cannot resolve the spatially varying, anisotropic morphology required for microstructure-based material modeling.

Although micro-scale approaches exist, they also rely on simplifications. Classical cell models describe diffusion-driven growth but neglect bubble interaction and deformation, while more advanced methods such as Lattice Boltzmann simulations are computationally expensive and often limited to idealized conditions [23,24]. Consequently, there remains a gap between efficient process simulation and detailed microstructure prediction.

### 1.3. Bubble-Growth Modeling at the Micro-Scale

To address this gap, a previous study introduced a micro-scale computational fluid dynamics (CFD) framework for FIM based on a two-phase, compressible volume-of-fluid formulation. In this approach, macro-scale pressure and temperature histories are used as boundary conditions for a local through-thickness domain, while bubble nucleation is calculated in a separate pre-processing step based on classical nucleation theory [27]. The generated bubble distribution is then mapped to the CFD model, enabling the investigation of local gas–melt interaction during, e.g., bubble elongation.

The model demonstrated that a resolved micro-scale description provides access to physical effects that are not captured by conventional macro-scale simulations. In particular, bubble deformation, shear-induced orientation, and coalescence could be observed directly, highlighting the interaction between melt flow, pressure gradients, and bubble evolution [27].

Similar multiscale approaches have also been proposed for plastic foaming processes in other contexts. For example, Geier and Piesche developed a coupled macro- and micro-scale model for polyurethane foaming, in which process-scale conditions are linked to a micro-scale description of foam evolution [28]. This work demonstrates the importance of multiscale modeling. However, its micro-scale description uses a phase-change model to depict the chemical reaction. Furthermore, the velocity, pressure, and temperature levels, as well as their temporal and spatial gradients are substantially lower than those encountered in injection molding.

Accurately modeling bubble growth remains a central challenge in microstructure-resolved simulations. Bubble growth in plastic foaming is typically described as a diffusion-driven process under conditions of thermodynamic supersaturation [29,30]. After nucleation, dissolved gas diffuses into existing bubbles due to local concentration gradients, leading to a coupled evolution of bubble radius, internal pressure, and surrounding gas concentration.

A large number of conventional bubble-growth models have been developed based on cell concepts, in which each bubble grows within a spherical domain of constant mass [29,31]. These models have been applied to extrusion and injection molding processes [32,33,34]. Extensions include the incorporation of viscoelastic effects [32,35], concentration-dependent diffusion and viscosity changes due to dissolved gas [29,36], and more advanced descriptions of the gas phase, such as the use of a van der Waals equation of state [30]. Furthermore, models combining viscoelastic constitutive equations with numerical methods such as the finite element method (FEM) have been proposed to capture the influence of rheological behavior on bubble growth [37,38].

Despite these developments, conventional models share fundamental limitations. They generally assume spherical bubble growth and neglect deformation under shear as well as interactions between neighboring bubbles, such as coalescence. In addition, bubble growth is typically calculated in isolated unit cells, preventing the direct simulation of spatially resolved morphology evolution. While these models can reproduce experimental trends, often using fitted parameters, they cannot capture the anisotropic and heterogeneous structures observed in injection-molded parts [29,30].

These limitations are also reflected in industrial simulation tools. For example, the bubble-growth model implemented in Moldex3D is based on cell-model assumptions, including spherical symmetry, constant diffusion coefficients, Newtonian melt behavior, and the absence of bubble interaction [22,39]. As a result, these approaches provide averaged quantities such as cell radius and density, but do not resolve the local morphology.

In contrast, the previously developed micro-scale CFD framework allows the direct simulation of bubble deformation, interaction, and coalescence. However, due to the absence of a physically based growth model, bubble expansion had to be approximated by assigning elevated initial pressures, leading to non-physical effects such as rapid growth and pressure oscillations [27]. The previous investigation also showed that bubble deformation is highly sensitive to the transferred pressure gradient. A reduction of the inlet-to-outlet pressure difference by approximately 50% produced a reduction of a similar magnitude in the simulated bubble aspect ratio [27]. This sensitivity highlights the need for consistency between the averaged macro-scale foaming model and the explicitly resolved expansion-driven flow at the micro scale.

Therefore, the realistic modeling of bubble growth remains essential for improving microstructure-resolved simulations. Classical diffusion-based approaches provide a physically grounded description of bubble evolution [31,40], while in thermoplastic systems the growth behavior is further influenced by rheological effects such as viscoelastic stresses and extensional viscosity [41,42].

The present work builds on the existing micro-scale framework and focuses on improving the description of bubble growth. The nucleation model, stochastic bubble placement, viscoelastic VoF formulation, and simulation of bubble deformation, interaction, and coalescence are adopted from the framework developed in [27]. The present study extends this framework by introducing a bubble-specific, diffusion-controlled mass-source formulation that is coupled iteratively to the evolving bubble geometry. By introducing a more physically consistent growth model, the aim is to eliminate the artifacts of the previous overpressure approach and evaluate whether the framework can reproduce experimentally observed qualitative trends in local foam morphology.

## 2. Materials and Methods

### 2.1. Bubble Growth Model

Bubble growth within the micro-scale CFD framework is described as a diffusion-controlled mass-transfer process from the surrounding plastic melt into the gas phase. The model is derived from the classical Rayleigh–Plesset equation, which is based on the conservation of momentum for a growing gas bubble embedded in a liquid medium [43,44]. The same relation can equivalently be derived from a balance of the pressures acting at the bubble interface [43,45].

In this formulation, the pressure inside the bubble is balanced by the surrounding forces, leading to:

(1) $$p_{gas}=p_{melt}+p_{Laplace}+p_{visc}+p_{dyn}$$

where $p_{melt}$ is the local pressure in the plastic melt, $p_{Laplace}$ is the capillary pressure due to surface tension, $p_{Visc}$ represents viscous pressure, and $p_{dyn}$ is a dynamic pressure contribution accounting for non-purely radial velocity fields [43,45].

In the present two-phase volume of fluid (VoF) OpenFOAM model, the pressure, viscous stresses, surface tension effects, and velocity field are resolved directly by the CFD solver [46]. Consequently, $p_{melt},\;p_{Laplace},p_{visc}\;and\;p_{dyn}$ are implicitly included in the momentum equation and do not need to be modeled separately. Therefore, the bubble-growth model focuses on the temporal evolution of the gas pressure $p_{gas}$, which is governed by the amount of gas transferred into the bubble.

Bubble growth is assumed to be governed by diffusion-controlled mass transfer of dissolved gas from the surrounding melt into the bubble. The driving force is the difference between the gas concentration in the bulk $C_{\infty }$ melt and the equilibrium concentration $C_{s}$ at the bubble–melt interface.

(2) $$\dot{m}=k\cdot A_{int}\cdot \left(C_{\infty }-C_{s}\right)$$

In the present implementation, $C_{\infty }$ is prescribed as a spatially uniform, time-dependent process input rather than calculated from a transported species-conservation equation. Accordingly, the local mass-transfer rate responds to the resolved bubble geometry, temperature, and gas pressure, whereas the temporal depletion of the bulk gas concentration is imposed externally.

This formulation represents a standard description of diffusion-controlled mass transfer $\dot{m}$ at a fluid interface $A_{int}$ and is consistent with classical bubble growth models such as Epstein–Plesset and cell-model approaches [32,40]. The mass transfer coefficient k depends on the diffusion coefficient D and convection.

For plastic melts, particularly after the filling phase, the Reynolds number is typically very small because of the high viscosity. Consequently, convective transport around the bubbles is neglected. In this diffusion-dominated regime, the mass transfer can be described using the Sherwood number for a sphere which corresponds to purely radial diffusion [32].

(3) $$Sh=\frac{k\cdot d}{D}=2\;\to k=\frac{2\cdot D}{d}$$

Here, d is the diameter of the bubble. However, the bubbles are sheared in the injection molding process, and the resulting non-spherical shapes have no explicit diameters. Therefore, a representative diameter is derived from the cross-sectional area of the 2D simulations.

(4) $$d_{rep}=2\sqrt{\frac{A_{cross}}{\pi }}$$

The gas concentration at the bubble interface is calculated using Henry’s law [47]. This is analogous to the nucleation model in previous works with a temperature-dependent solubility coefficient described by a van ’t Hoff-relation:

(5) $$C_{s}=S\left(T\right)\cdot p_{gas}$$

The diffusion coefficient is modeled as a temperature-dependent quantity using an empirical power–law relation:

(6) $$D(T)=D_{0}{\left(\frac{T}{T_{D0}}\right)}^{n}$$

This simplified description captures the dominant temperature influence while neglecting potential dependencies on pressure and concentration.

### 2.2. Implementation of the Bubble Growth Model

The equations introduced in Section 2.1 are implemented in a Python (version 3.11.9) script to calculate the time-dependent mass source for each individual bubble. The implementation of the mass flow into the CFD model is achieved using the OpenFOAM function massSource.H [46]. This function enables the definition of spatially resolved mass sources or sinks within the fvModels framework for selected cells or cell sets. In the present approach, the calculated gas mass transfer is assigned as localized source terms at the bubble centers along their trajectories. In this way, the diffusion-driven increase in gas mass can be introduced into the gas phase without modifying the underlying two-phase flow solver.

The CFD simulations are performed using the micro-scale OpenFOAM model introduced in the previous work [27]. The model is based on a compressible two-phase volume-of-fluid formulation, which resolves the gas and plastic melt phases through a phase-fraction field. The interface between both phases is captured using the VoF method, while surface tension is represented by the continuum surface force formulation. The pressure–velocity coupling is solved with the PIMPLE algorithm, and the gas phase is treated as compressible. The plastic melt is described as a shear-thinning, viscoelastic fluid using a multi-mode Phan–Thien–Tanner constitutive model. The model therefore resolves the coupled evolution of pressure, velocity, temperature, phase distribution, and viscoelastic stresses. As shown in the previous study, this framework allows the direct simulation of bubble transport, deformation, shear-induced orientation, and coalescence within a local through-thickness domain [27].

In the present work, the fluid-mechanical part of the OpenFOAM model remains unchanged. Consequently, pressure gradients, viscous stresses, surface tension forces, bubble deformation, and flow-induced bubble motion are still calculated directly by the CFD solver. The newly introduced Python-based model does not prescribe bubble growth through an artificial pressure increase but instead calculates the additional gas mass entering each bubble from the surrounding plastic melt. The resulting mass source changes the gas mass locally and thereby modifies the internal bubble pressure through the gas equation of state used by the compressible solver. This provides the coupling between diffusion-controlled gas uptake and the resolved two-phase flow field.

The Python script uses files in the visualization toolkit (VTK) format exported from the OpenFOAM simulations to identify and characterize individual bubbles. For each time step, the phase-fraction field is evaluated using connected-component labeling to identify spatially connected gas regions, which are interpreted as individual bubbles [48]. This approach is standard in multiphase CFD analysis and provides an unambiguous partitioning of the domain into discrete bubble objects [49].

For each detected bubble, the projected two-dimensional area A_cross_, interfacial area A_int_, and centroid position are calculated. Since the simulation domain is a thin 2D structure, bubble cross-sections are projected onto the x-y plane and analyzed using 2D convex hull decomposition to determine the boundary polygon [50]. The 2D convex hull area is then computed using SciPy’s standard ConvexHull function [49], yielding the effective bubble cross-sectional area in the horizontal plane. This projected cross-sectional area is the area occupied by the bubble in the two-dimensional computational x-y plane and is used to evaluate bubble size and growth.

The bubble perimeter is calculated as the sum of edge lengths along the convex hull boundary in 2D. The interfacial surface area between gas and melt is then estimated as the perimeter multiplied by the extrusion thickness, following the assumption of a uniform thin layer. This provides the area available for mass transfer across the bubble–melt interface.

The geometric center of each bubble is determined as the mean position of all points belonging to the connected gas region, providing a representative location for source-term placement in the mass transfer model. To obtain time-dependent mass transfer rates, bubbles are tracked across consecutive time steps by identifying corresponding gas regions based on spatial proximity and geometric continuity. This tracking procedure reconstructs the trajectory of each bubble through the melt domain. Local bubble properties and surrounding process conditions are then evaluated at each time step and used to calculate the instantaneous mass-transfer rate according to the governing equations presented in Section 2.1.

### 2.3. Iterative Coupling Workflow

**Figure 1:**
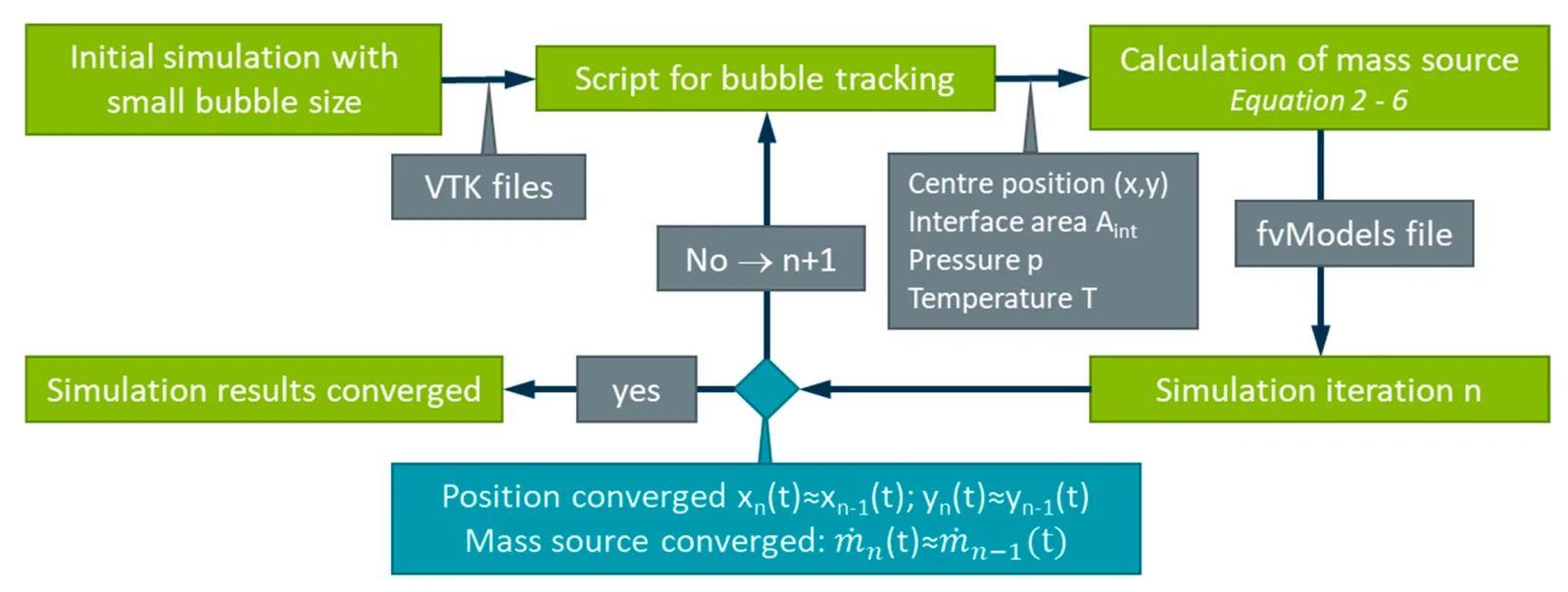
Iterative workflow of calculating the mass source and simulating the bubble growth and deformation.

The overall simulation procedure follows an iterative coupling workflow (Figure 1). First, bubble nucleation is calculated according to the approach introduced in the previous work [27]. The nucleation model is adopted without modification from the previous work and is based on classical nucleation theory. The local homogeneous and heterogeneous nucleation rates are calculated from the pressure, temperature, and dissolved-gas concentration fields. The resulting total nucleation rate is integrated over the prescribed nucleation interval to determine the expected number of nuclei within the micro-scale domain.

The bubble positions are generated using a non-homogeneous Poisson point process. For this purpose, the local nucleation rate and cell volume define a spatial probability distribution over the computational mesh. Cells are selected stochastically according to this distribution, while a geometric overlap check prevents newly initialized bubbles from intersecting existing bubbles. Following each successful placement, the local dissolved-gas concentration and consequently the nucleation probability in the surrounding region are reduced to account approximately for gas depletion. A temperature-based criterion further excludes positions at which the local thermal history would not permit the bubble to reach a mesh-resolvable size before stabilization. The complete equations and implementation details of the nucleation and placement algorithms are provided in [27].

In contrast to the earlier model, however, the initial bubble radius is not chosen to emulate later bubble growth. Instead, the bubbles are initialized with the minimum initial bubble radius selected for the present mesh resolution. This reduces the artificial influence of the initial condition and allows the subsequent growth to result from the calculated mass transfer.

After the initial bubble distribution has been generated, a first OpenFOAM simulation is performed without bubble growth. In this initial run, the bubbles are transported by the melt flow caused by the pressure gradient and deform according to the local velocity and stress fields. The purpose of this simulation is to obtain a first estimate of the bubble trajectories, center positions, cross-sectional areas, and interfacial surfaces. The resulting VTK files are then processed by the Python script, which calculates the corresponding mass source terms for each bubble over time.

In the next step, a second OpenFOAM simulation is conducted using the calculated mass sources (Figure 1). Due to the added gas mass, the bubbles grow during the simulation. This growth changes the local gas volume, the interfacial surface, the bubble velocity, and in some cases the bubble center position. Since all these quantities influence the mass transfer rate, the mass sources calculated from the initial simulation are only a first approximation. Therefore, the resulting VTK files are again exported and processed with the Python script to update the bubble trajectories and mass source terms.

The iterative procedure was continued until the relative changes in the total transferred gas mass and final mean bubble area between two successive coupling iterations were below a 1%tolerance. Once convergence is reached, the coupled workflow provides a consistent solution in which bubble growth, bubble motion, and local mass transfer are mutually adapted.

### 2.4. Demonstration Use Case

**Figure 2:**
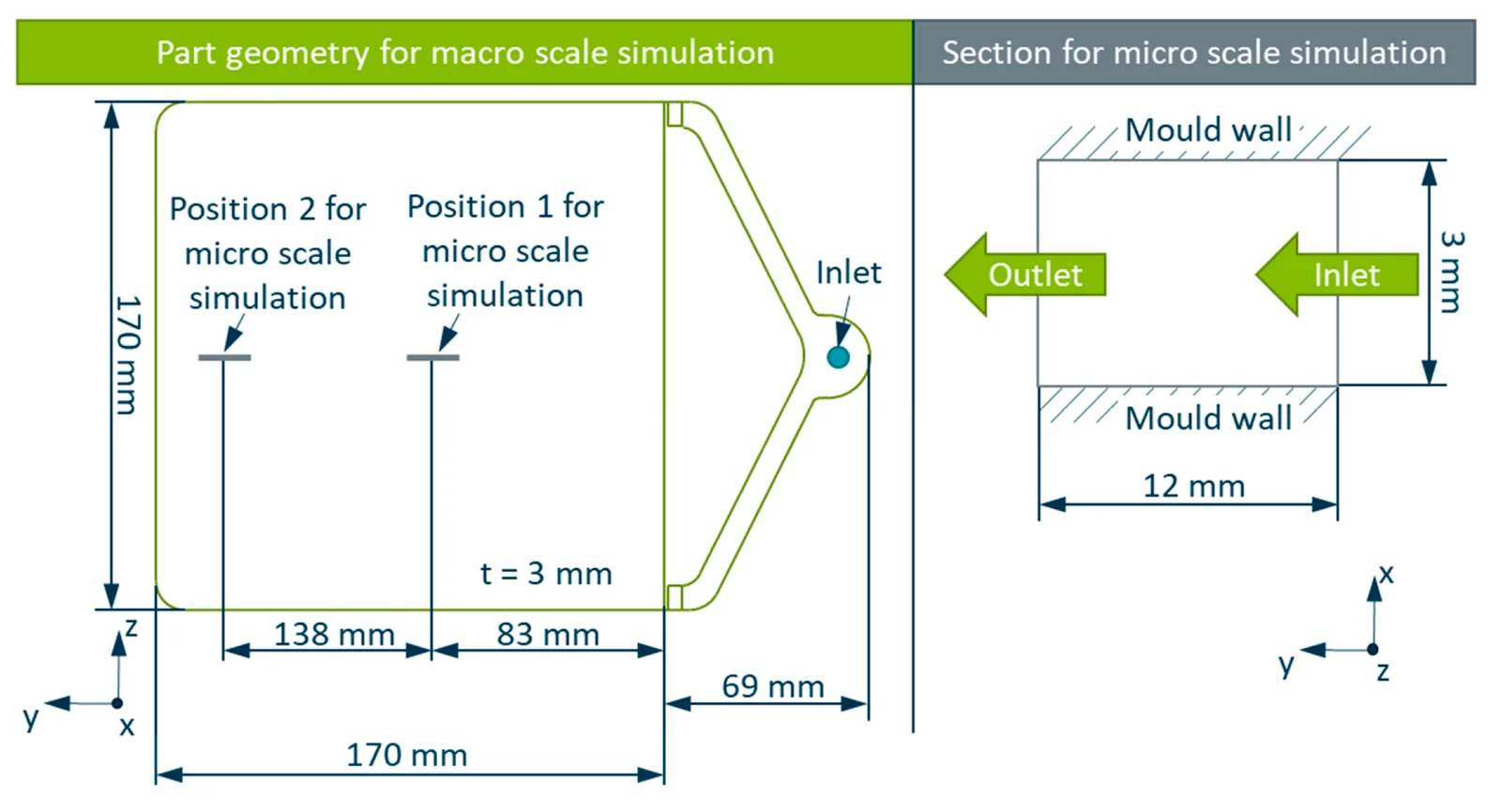
Part geometry to produce foamed thermoplastic samples, the sampling positions for microscopy images and location of the micro-scale simulation as well as the inlet/outlet modeling of the micro-scale domain.

The proposed approach was applied to an injection-molded plate geometry, shown in Figure 2, to evaluate the micro-scale simulation workflow. The plate was produced from polycarbonate (PC) LED 5102 (Covestro AG, Leverkusen, Germany) using a SmartPower 240/1330 injection molding machine equipped with the CELLMOULD process (WITTMANN BATTENFELD GmbH, Kottingbrunn, Austria). Nitrogen (N_2_) was injected into the plastic melt during the dosing phase. Two process parameter sets with different melt temperatures, mold temperatures, and injection rates were investigated (Table 1). The parameter sets were selected to produce distinctly different cell morphologies under low-pressure foam injection molding conditions.

Three samples were extracted for each process parameter set from each position indicated in Figure 2 and embedded in resin before polishing. Cross-sectional images in the z-direction were acquired using a VHX-X1 digital microscope (Keyence Corporation, Osaka, Japan). The cell position, size, aspect ratio, and orientation were evaluated with ImageJ (version 1.54d) using the EllipseSplit plugin [51]. The images were calibrated using a spatial scale of 1.05 μm/pixel, resulting in an analyzed field of view of 2.997 × 16.248 mm^2^. The cell phase was separated from the surrounding plastics using a global gray-value threshold of 0.5. Detected features with a major axis length below 16 µm were excluded to remove image noise and polishing artifacts. Cells intersecting the boundaries of the analyzed image were excluded because their complete geometry and centroid position could not be determined. The same image-processing procedure and filtering criteria were applied to all samples.

**Figure 3:**
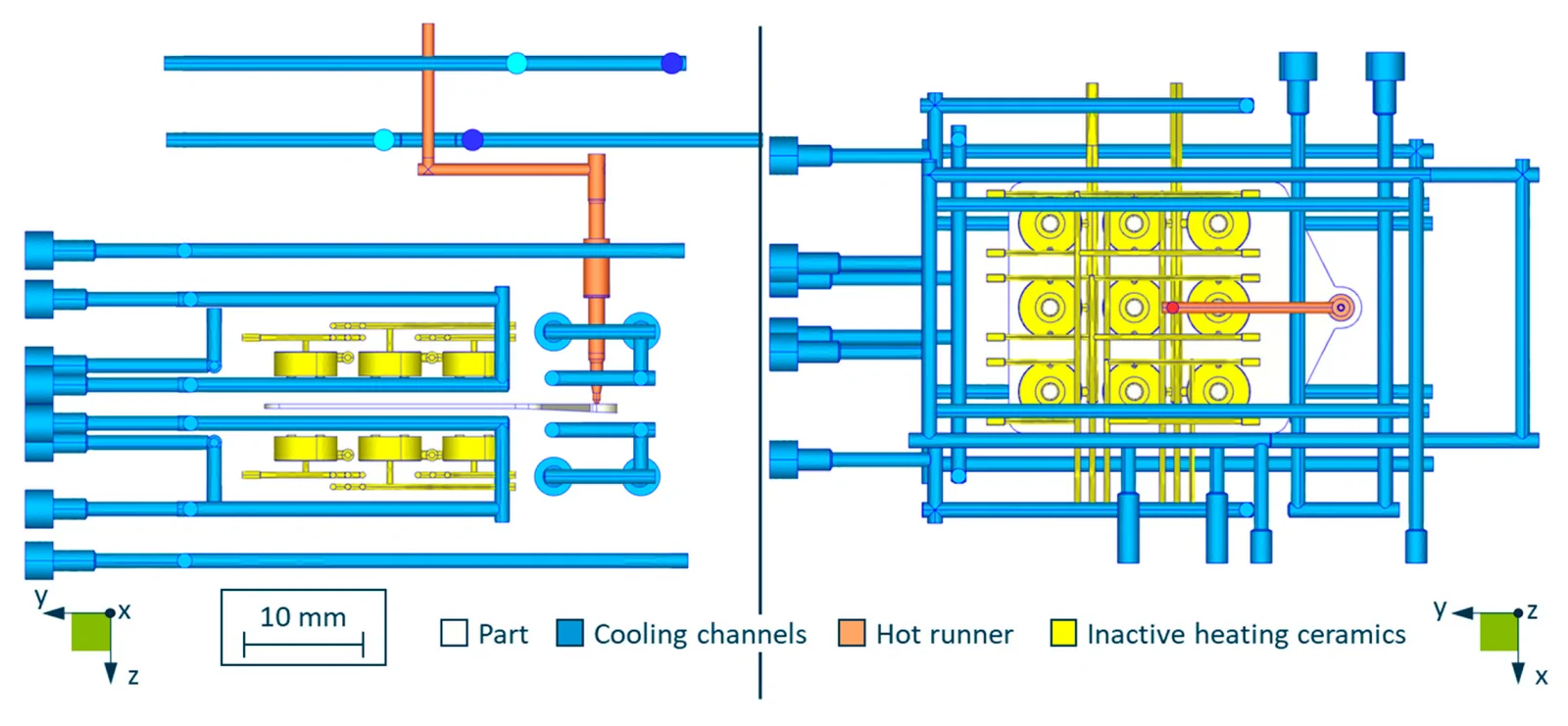
Model setup of the macro-scale simulation, including the part cavity, cooling channels, hot runner, and inactive heating elements [27].

Analogously to the experiments, the part geometry and process parameters listed in Table 1 were used for macro-scale FIM simulations with Moldex3D 2024 (CoreTech System Co., Ltd., Taiwan). The simulation model, including the part cavity, cooling channels, hot runner, and inactive heating elements, is shown in Figure 3. Foaming at the macro scale was calculated using the Han and Yoo model with the Moldex3D standard parameter set for N_2_ (Table 2) [52].

The material properties of the plastic melt used in both the macro-scale and micro-scale simulations are listed in Table 3. The same basic material data were used in both simulation levels. However, the viscosity was described by a Cross-WLF model in the macro-scale Moldex3D simulation, whereas the micro-scale OpenFOAM model used the Phan–Thien–Tanner (PTT) formulation [27]. The shear-viscosity curves of both models are comparable over the shear-rate and temperature ranges relevant to the present simulations, whereas the PTT model additionally represents viscoelastic and extensional stresses. The material parameters of N_2_, including the solubility and diffusivity parameters used in the micro-scale simulation, are listed in Table 4.

In the micro-scale model, the foaming parameters from the macro-scale simulation were extended to include temperature-dependent diffusion coefficient, solubility, and surface tension. The temperature dependence of the solubility was described by an Arrhenius-type relation, whereas the diffusion coefficient was described by a power–law relation [47]. The surface tension was described by a linear temperature correlation:

(7) $$\sigma (T)=\sigma_{ref}\left(1-k_{\sigma }\cdot (T-T_{\sigma ,ref})\right)$$

From the two macro-scale simulations, each corresponding to one process parameter set, the local pressure and temperature histories were extracted at the two positions shown in Figure 2 and transferred as boundary conditions to the micro-scale simulations. The extracted pressure histories, units, and spatial positions were verified against the original Moldex3D results, excluding an error during data export or conversion. However, directly applying the macro-scale pressure difference resulted in melt velocities and bubble deformations that substantially exceeded the experimental observations. Previous investigations showed that bubble deformation is highly sensitive to pressure-driven flow. Reducing the inlet-to-outlet pressure difference by approximately 50% produced a reduction of a similar magnitude in the simulated bubble aspect ratio. Later bubble initialization reduced deformation because the pressure gradient declined over time [27].

For the present investigations, the inlet-to-outlet pressure difference was therefore reduced to 10% of the macro-scale value. The factor of 0.10 was selected iteratively by reducing the transferred pressure difference until the simulated bubble deformation was of the same order as that observed experimentally, while retaining the pressure-driven flow direction toward the unfilled cavity region. This case-specific correction was applied to establish micro-scale flow conditions that produced physically plausible cell deformation and is not considered a transferable material or coupling parameter. The bubbles were initialized at 2.0 s for process parameter set A and at 2.5 s for process parameter set B. The implications of this correction for the one-way macro-to-micro coupling are discussed in Section 4.2. The micro-scale simulation domain shown in Figure 2 was discretized using blockMesh with a uniform element size of 40 µm × 40 µm. The micro-scale simulation procedure described in Section 2.3 was then applied using the material parameters listed in Table 3 and Table 4. In the present simulations, all nucleated bubbles were introduced during one nucleation event with an initial radius of r_0_ = 60 µm. The corresponding initial diameter of 120 μm120\,\mu\text{m}120 μm spans three computational cells and defines the minimum bubble size represented with the present mesh. Although this value is considerably larger than the critical nucleation radius predicted by classical nucleation theory, it represents a mesh-resolvable bubble size. The initialized bubbles are therefore interpreted as nuclei that have already undergone an unresolved early growth stage after nucleation rather than as bubbles at the thermodynamic critical radius [27]. The resulting bubble populations at Positions 1 and 2 for process parameter set A and set B are listed in Table 5. These values result from the local nucleation probabilities and the geometric and thermal placement criteria. Smaller nuclei and bubbles that are not expected to reach a size which can be depicted by the mesh resolution are excluded from the resolved simulation. Consequently, the mesh and initialization procedure are not intended to represent the lower end of the experimental cell-size distribution.

**Figure 4:**
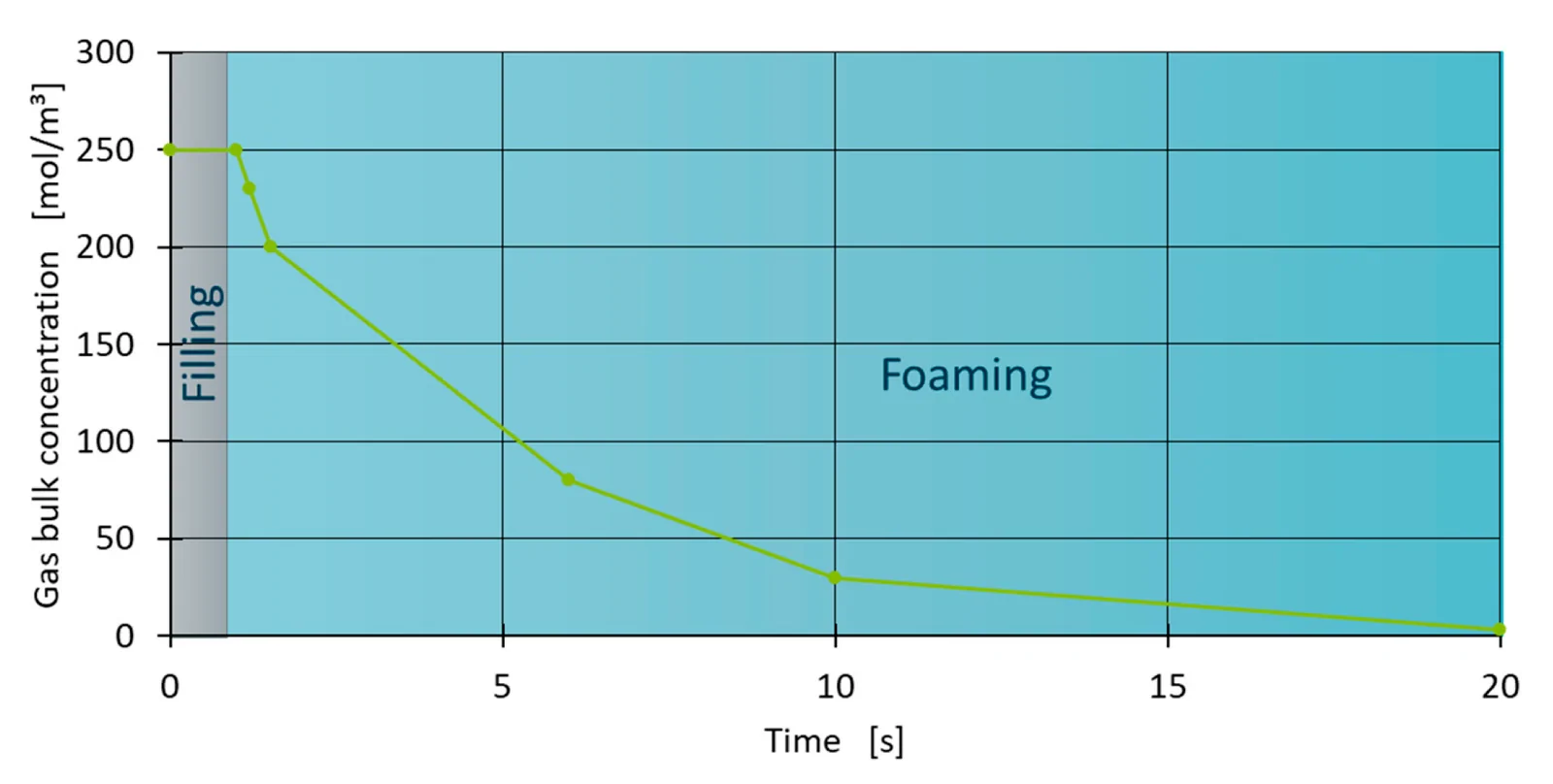
Bulk gas concentration development used as input for the mass-flow calculation.

In addition, a simplified, spatially uniform bulk gas concentration history was prescribed for the mass-transfer calculation at both sampling positions (Figure 4) [55]. The history was not fitted to the resulting cell morphology. It is bounded by the initial gas concentration of 0.6 wt% and the final equilibrium concentration and decreases monotonically to represent consumption of dissolved gas during nucleation and bubble growth. The decreasing slope represents the diminishing concentration driving force as equilibrium is approached. The same concentration-history formulation was used for all investigated simulations. Because the concentration is prescribed rather than obtained from a species-conservation equation, the model does not predict the global or spatially resolved dissolved-gas balance.

The simulations employed adaptive time stepping with an initial time-step size of 1 × 10^−8^ s and a maximum time-step size of 2 × 10^−3^ s. The time step was constrained by maximum flow and phase-fraction Courant numbers of maxCo = 0.15 and maxAlphaCo = 0.15, respectively. Pressure–velocity coupling was performed using the PIMPLE algorithm with three outer correctors, three pressure correctors, and no non-orthogonal correctors. The phase-fraction equation used one correction and two sub-cycles, with five limiter iterations.

The following section first evaluates the functionality of the implemented bubble-growth model and then compares the predicted spatial cell morphology with the experimental results for both process parameter sets and sampling positions.

## 3. Results

### 3.1. Evaluation of Bubble-Growth Model

**Figure 5:**
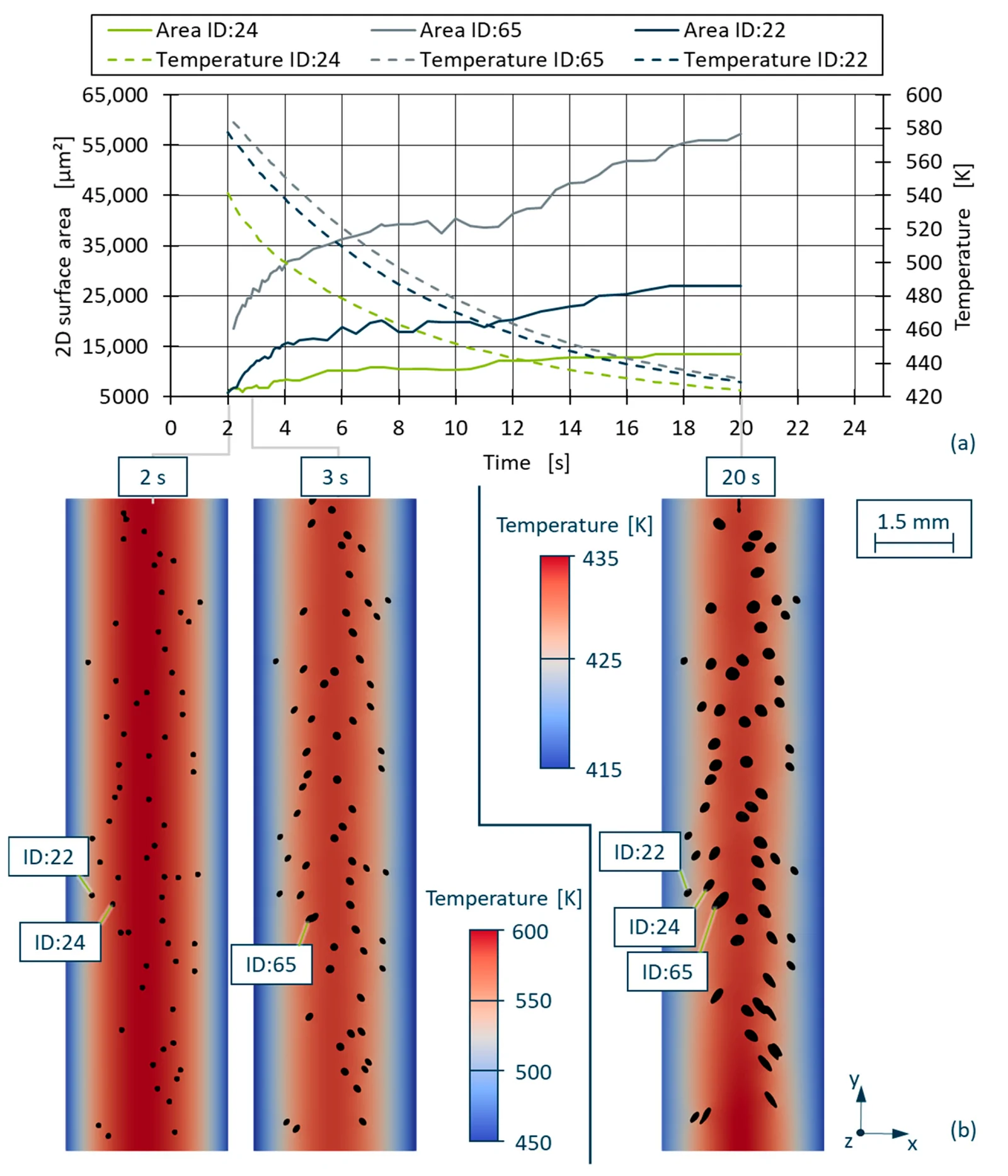
Development of the projected two-dimensional bubble area and temperature of three selected bubbles (**a**), and temperature distribution with bubble geometries shown in black for process parameter set A (Table 1) at Position 1 (Figure 2) after 2 s, 3 s, and 20 s (**b**). The temperature scale is adjusted for the distribution at 20 s.

To evaluate the functionality of the implemented cell growth model, the bubble morphology predicted by the micro-scale simulation was analyzed for Position 1 (Figure 2) and process parameter set A (Table 1). Figure 5a shows the temporal evolution of the projected two-dimensional area of three selected bubbles. The selected bubbles have identical initial sizes but are located at different positions across the thickness and therefore at different distances from the cavity wall. As a result, their growth behavior differs due to the local temperature and viscosity fields. Bubbles located closer to the wall experience lower temperatures and, consequently, higher melt viscosity, which increases the resistance against cell growth. According to Equation (1), this higher viscous resistance reduces the local growth rate for otherwise comparable initial conditions. In addition, larger bubbles provide a larger gas–melt interface area, resulting in a higher gas mass flow rate according to Equation (2) and therefore faster subsequent growth. This also means that the initial bubble radius after nucleation influences the result. However, the effect of the local temperature and pressure history dominates the resulting cell size.

**Figure 6:**
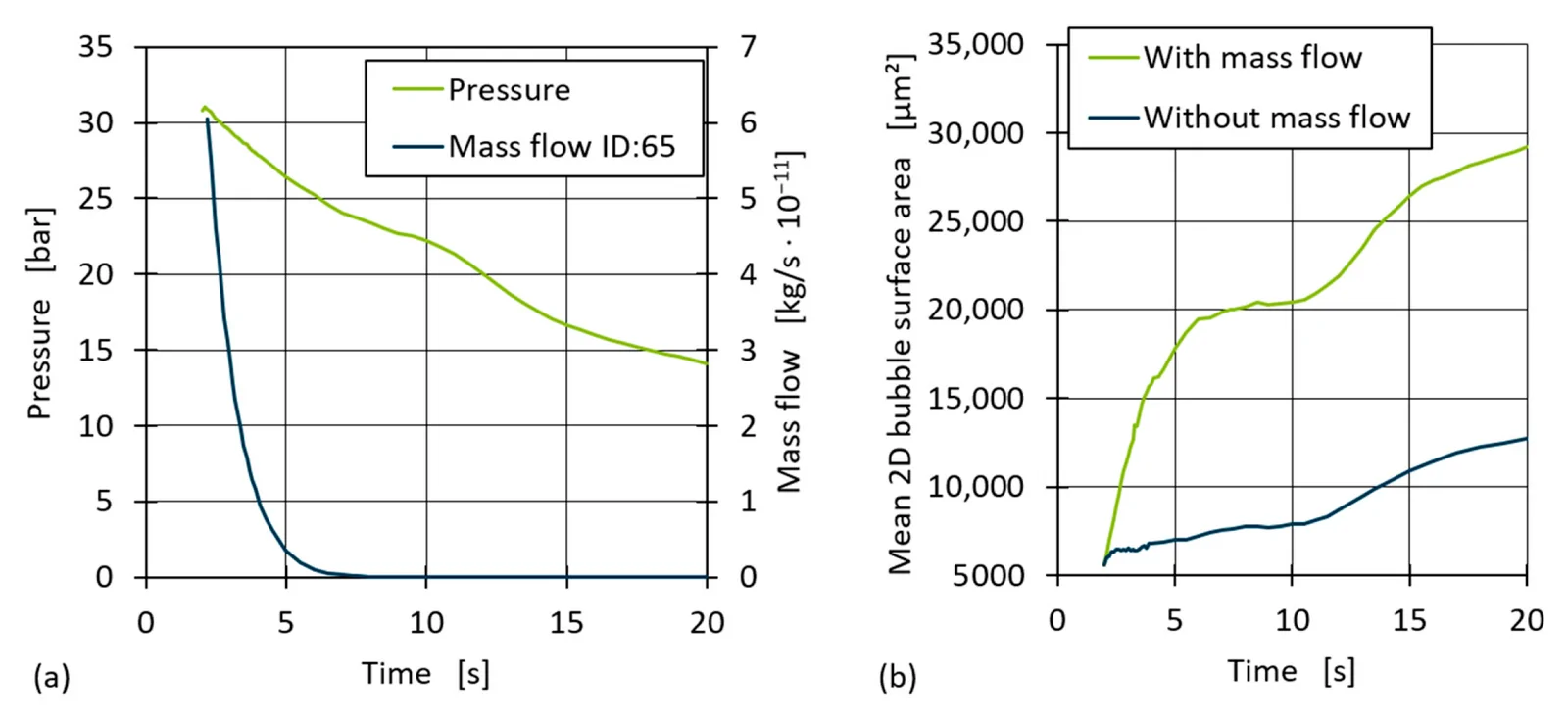
Simulated pressure development and calculated average gas mass flow rate (**a**), and development of the average projected two-dimensional bubble area with and without the cell growth model (**b**) for process parameter set A (Table 1) at Position 1 (Figure 2).

The increase in bubble size is caused by the modeled gas mass flow into the bubbles and by the decreasing melt pressure, as shown in Figure 6a. The calculated mass flow becomes negligibly small after approximately 6 s, corresponding to about 4 s after nucleation. After this point, further bubble growth is mainly driven by the decreasing melt pressure. Since the pressure dependence of the melt specific volume is small compared with the ideal gas behavior of the gas phase, the remaining growth is largely governed by the ideal gas law. This pressure-driven contribution is reduced by the increasing melt viscosity and decreasing temperature during cooling.

Due to this approximately proportional relation between gas volume and pressure, the absolute pressure-driven growth contribution is larger for already larger bubbles than for smaller ones. If the diffusion-driven mass flow is not included in the micro-scale simulation, the predicted average bubble area remains significantly smaller (Figure 6b).

Overall, the calculated gas mass flow accounts for the major part of the bubble area increase during the initial growth period. In contrast to the surrogate overpressure approach used previously [27], the present simulations show continuous and monotonic bubble growth without pronounced pressure or velocity oscillations. The resulting morphology contains larger bubbles in the core and smaller bubbles toward the cavity wall. A comparison of these spatial trends with the experimental results is presented in Section 3.2.

### 3.2. Comparison of Simulated and Experimental Results

**Figure 7:**
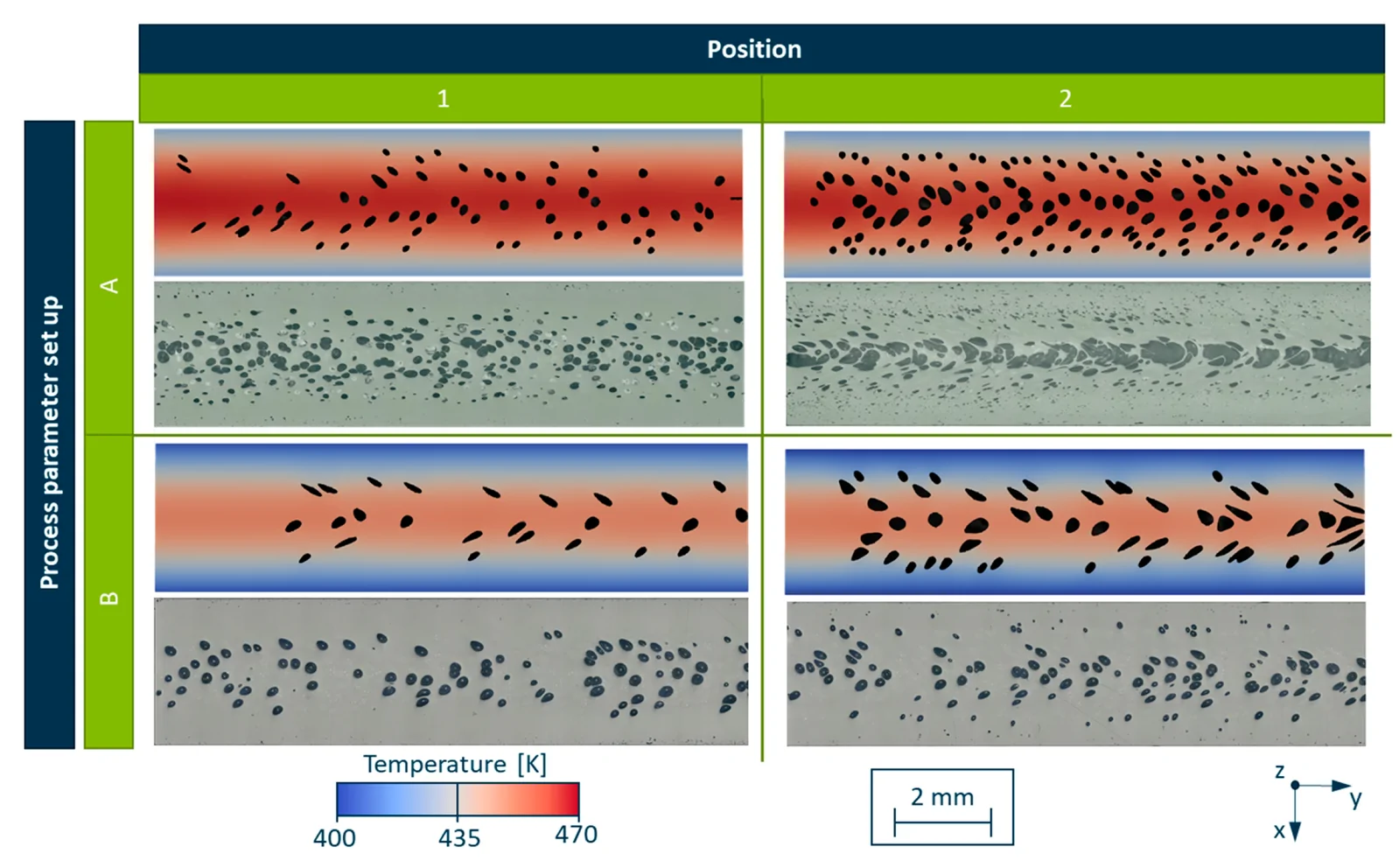
Representative experimental cross-sections and simulated bubble distributions for Positions 1 and 2 and process parameter sets A and B. Bubbles are shown in black, and the simulated background indicates the temperature distribution.

**Figure 8:**
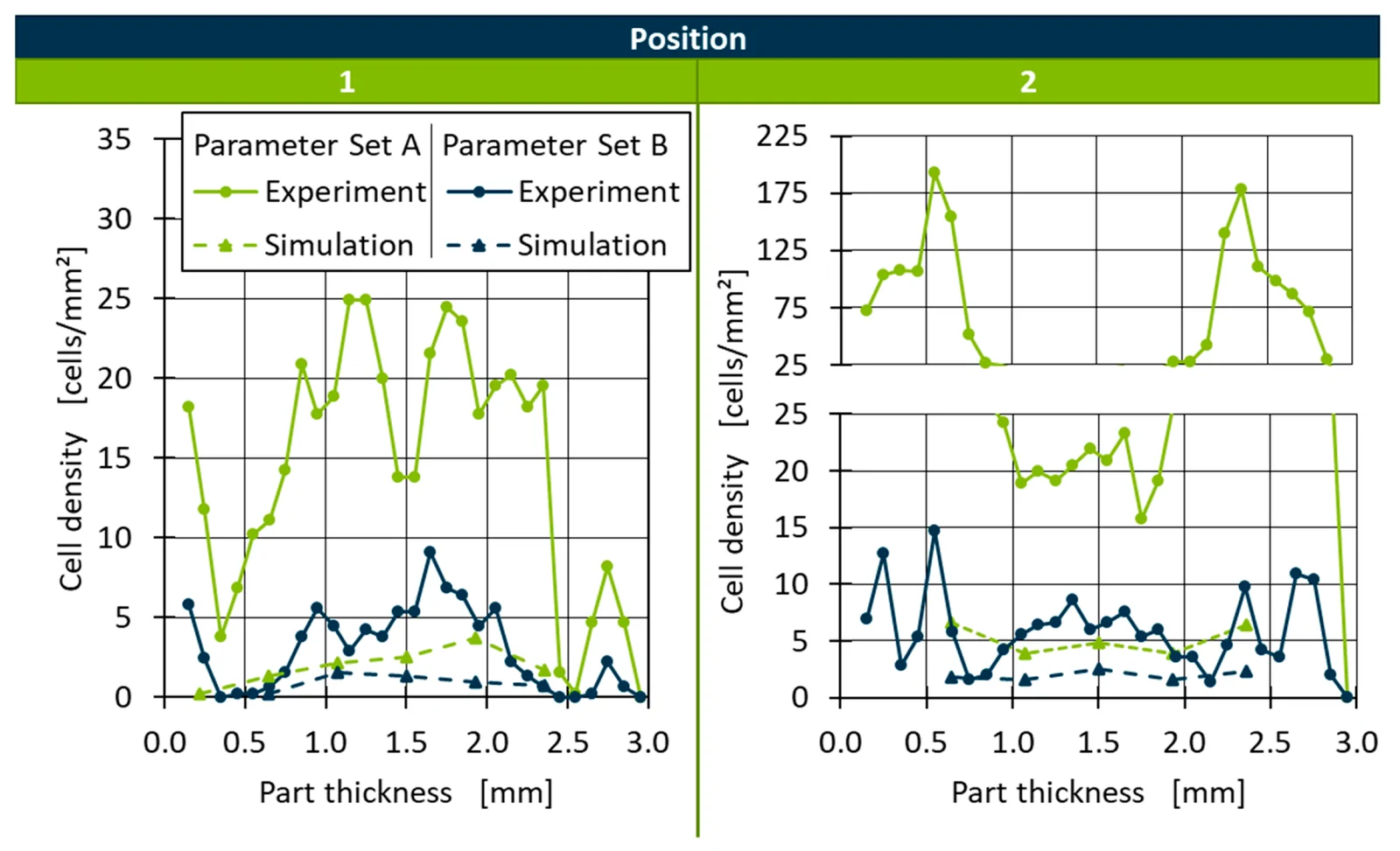
Comparison of experimental and simulated cell density distribution at different positions and process parameter sets.

To assess the capability of the micro-scale model to reproduce experimentally observed morphology trends, the simulation results were compared with experimental cross-sectional images. The comparison evaluates whether the model qualitatively reproduces the effects of different pressure, temperature, and shear histories on the resulting cell morphology. Figure 7 presents the simulated bubble distributions and representative experimental cross-sections for each position and process parameter set. Figure 8 shows the corresponding areal cell density distributions across the part thickness.

The final cell count is determined by the competing processes of nucleation and coalescence. The experimentally determined areal cell density is expected to exceed that of the two-dimensional simulation because the polished cross-section intersects cells whose centers are located outside the analyzed plane. In contrast, nucleation in the simulation is restricted to the computational plane. Although the absolute values are therefore not directly equivalent, the relative effects of the process parameters on nucleation and coalescence can still be compared. Accordingly, the cell-density comparison is used to evaluate relative spatial and process-dependent trends rather than quantitative agreement between the absolute values. Both the experiments and simulations show a higher cell density for process parameter set A, which uses higher melt and mold temperatures.

At Position 1, both the simulated and experimental results show a higher cell density in the core than in the adjacent shear layer. The simulated distribution does not reproduce the experimental cell population in the frozen surface layer because cells below the mesh-resolvable size are not initialized. Coalescence also affects the distribution. For process parameter set A at Position 1, the experimental cell density decreases locally in the core, where the larger cell size and smaller cell spacing increase the probability of coalescence. This reduction is less pronounced in the two-dimensional simulation because its lower in-plane cell density produces larger distances between neighboring cells.

**Figure 9:**
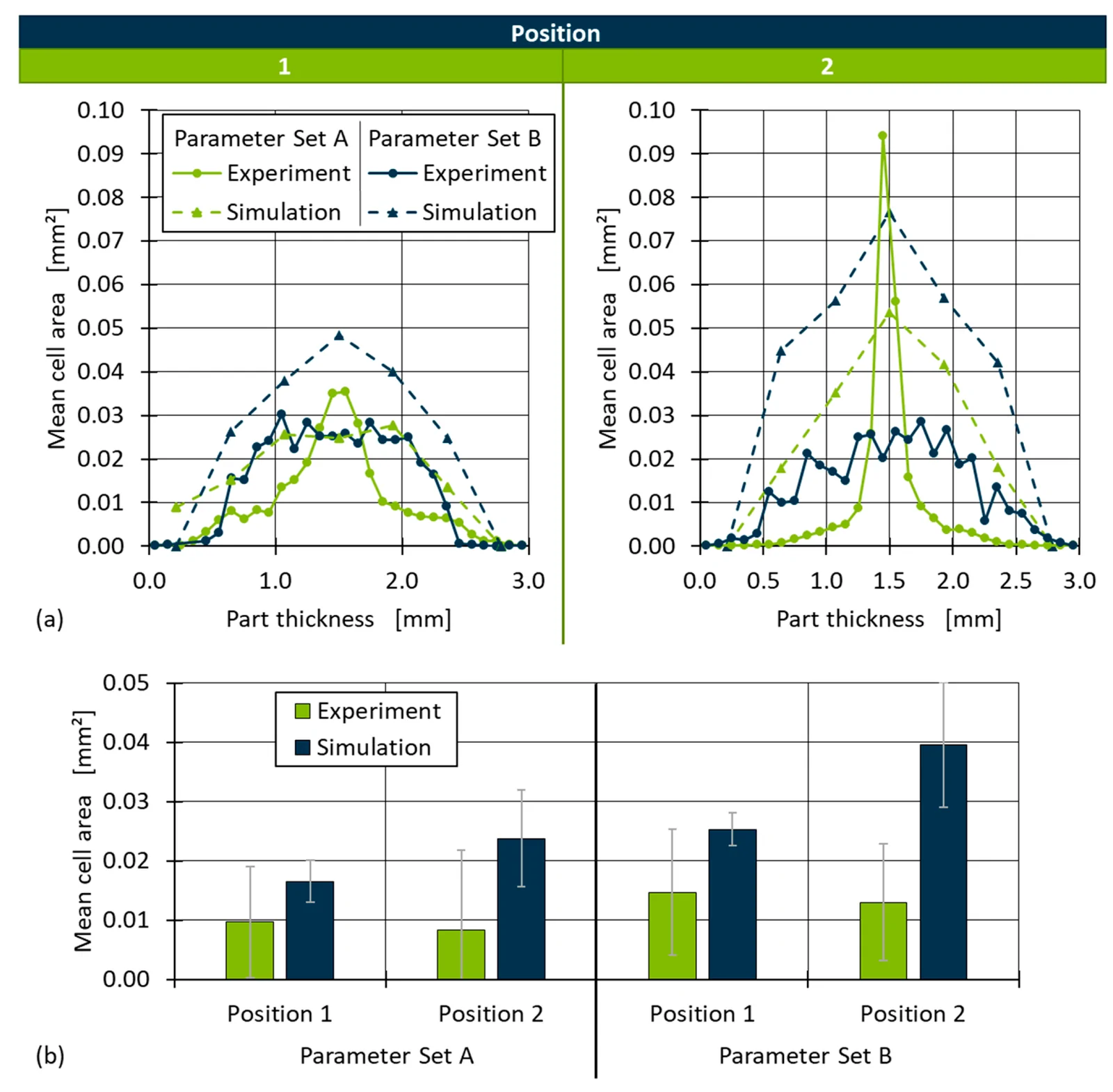
Comparison of experimental and simulated average cell area distribution (**a**) as well as overall average cell area (**b**) at different positions and process parameter sets.

The modeled bubble growth is analyzed by the cell size distribution over the part thickness. Figure 9 shows the average cross-sectional cell area for the different positions and process parameter sets. Both the experimental and simulated results show the characteristic through-thickness distribution, with larger cells in the core and decreasing cell size toward the cavity walls [7,56,57]. At the compact surface layer, the cell area converges toward zero, because the real parts only have a few very small cells and the simulation results show no bubbles as these small cell sizes are not initialized. The simulation therefore accounts qualitatively for the effects of the lower near-wall temperature, including higher melt viscosity, greater resistance to bubble growth, and earlier cell stabilization.

The simulation results show distinguishable differences in the distribution of the cell area between the two positions and parameter sets. The position further away from the inlet (Position 2) shows an overall higher cell area. For process parameter A, the mean cell area across the part thickness increases from 0.017 mm^2^ at Position 1 to 0.024 mm^2^ at Position 2. For parameter set B, it increases from 0.025 mm^2^ to 0.04 mm^2^. The simulation therefore predicts a substantial increase along the flow path (Figure 9b). This trend is plausible for a short-shot FIM process because the pressure level and pressure development are lower at the end of the flow path. The resulting resistance to bubble growth is therefore lower than at positions closer to the inlet. However, the experimental results show a more similar cell area closer to the inlet and further away from it. There is even a slight decrease along the flow path. Consequently, the simulated difference in average cell area between Positions 1 and 2 is larger than the experimentally observed difference.

Although the model generally overestimates the cell area, it reproduces the qualitative direction of the process-parameter influence. Both the experimental and simulated results show a smaller mean cell area for process parameter set A than for parameter set B. The higher cell density obtained for process parameter set A (Figure 8) distributes the available gas among a larger number of cells, whereas, for process parameter set B, the available gas is distributed among fewer cells, resulting in a larger average cell area. The only exception is the center of set A where the average cell area increases sharply and exceeds the local average cell area of set B due to the many coalescence events. This is not depicted by the simulation due to the previously explained fewer bubbles in a 2D simulation.

**Figure 10:**
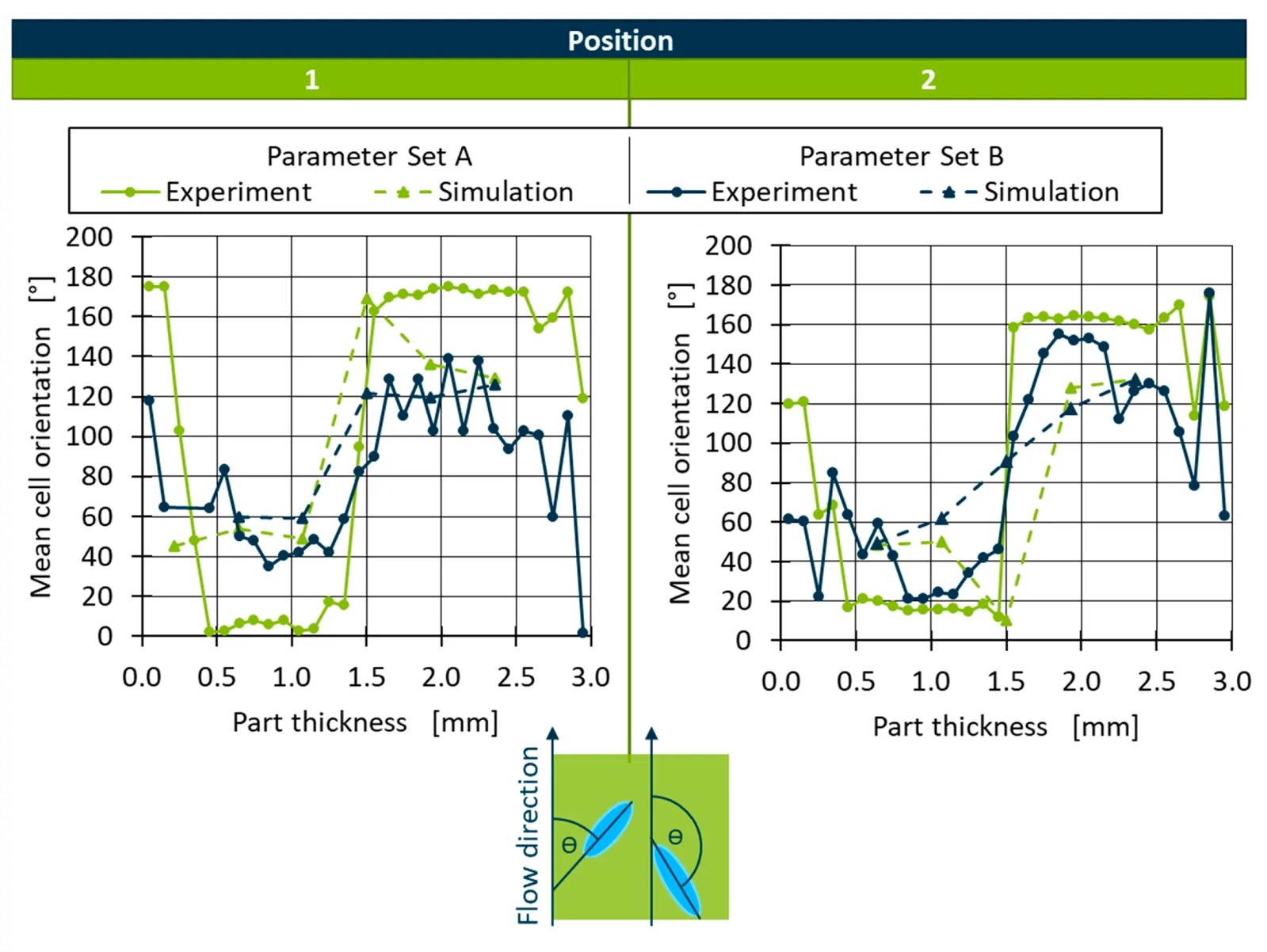
Comparison of the experimental and simulated mean cell orientation across the part thickness at different positions and for process parameter sets A and B.

**Figure 11:**
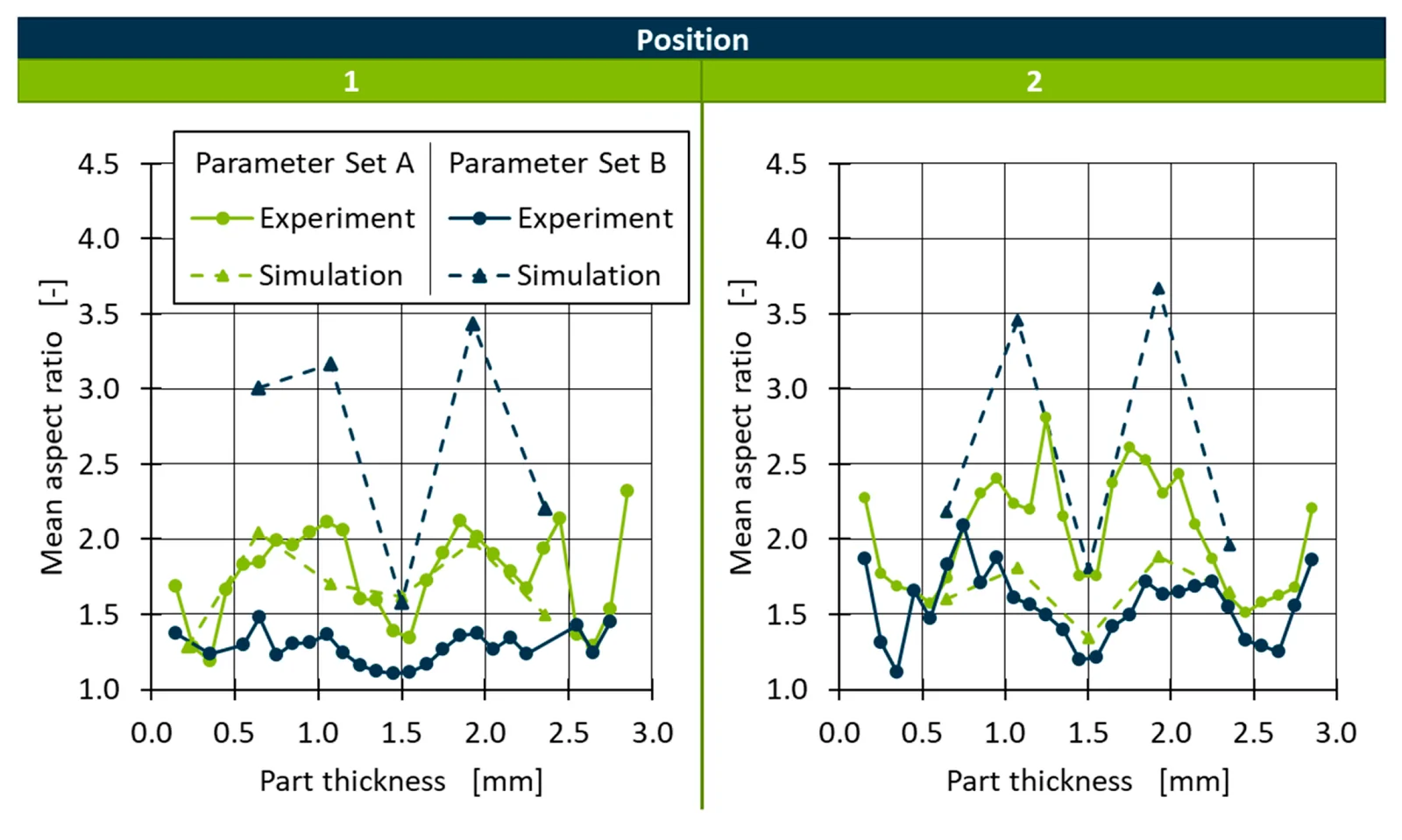
Comparison of the experimental and simulated mean cell aspect ratio across the part thickness at different positions and for process parameter sets A and B.

Bubble deformation results from the combined effects of shear flow, bubble growth, melt rheology, and surface tension. Figure 10 and Figure 11 compare the resulting cell orientation and aspect ratio across the part thickness. Both the simulated and experimental morphologies show cells aligned preferentially with the flow direction in the shear layer between the core and surface regions. In the core, the lower shear rate produces less pronounced alignment, whereas cells in the experimental surface layer tend toward an orientation of approximately 120 to 170 ° and 10 to 50 ° on the other half. This near-surface behavior is attributed to the rapid cooling and early stabilization of the small cells. Moreover, the increased Laplace pressure associated with a smaller cell radius provides greater capillary resistance to deformation.

The simulations reproduce the qualitative through-thickness trend in cell orientation. However, for process parameter set A, the experimental cells show substantially stronger preferential alignment with the flow direction in the shear and near-surface regions than the simulated cells and parameter set B. Two possible reasons are discussed in Section 4.2.

The experimental orientation data also exhibit considerable scatter. This is partly attributable to the larger number of cells intersected by the experimental cross-section and partly to the uncertainty associated with determining the major-axis orientation of cells with aspect ratios close to unity. For nearly circular cells, small variations in the detected contour can cause substantial changes in the calculated orientation angle.

The comparison of the aspect ratio provides further information on the magnitude of cell deformation (Figure 11). The experimental cross-sections contain a considerable proportion of small features with aspect ratios close to unity. These features include cells formed by secondary nucleation, which have less time to grow and deform, as well as off-center intersections of three-dimensional cells that appear smaller and more circular in the polished cross-section. Both effects reduce the experimentally determined mean aspect ratio. By contrast, nucleation in the present two-dimensional simulation is introduced over a limited time interval, and each simulated cell is evaluated in the plane containing its center. The simulations therefore do not reproduce the full population of small, late-nucleated cells or the sectioning effects present in the experimental measurements.

Nevertheless, the simulation reproduces the characteristic through-thickness tendency of the aspect ratio, with stronger deformation in the shear layer and lower aspect ratios in the core and near the rapidly cooled surface region. For process parameter set A, the simulated aspect ratios are predominantly between 1.5 and 2.0 and are therefore comparable to the experimentally determined values. For process parameter set A, this agreement indicates that the model captures the characteristic spatial trend and approximate magnitude of cell deformation. However, the larger deviations obtained for parameter set B show that this agreement cannot yet be generalized as quantitative validation of the deformation model. The remaining quantitative differences, particularly for process parameter set B, may arise from uncertainties in the temperature-dependent viscosity and cooling conditions. Furthermore, the simulated velocity field results from both the prescribed pressure gradient and the displacement of melt caused by bubble expansion. The pressure difference establishes the net flow direction toward the unfilled part of the cavity, whereas bubble growth locally modifies the magnitude and spatial distribution of the flow.

Table 6 summarizes the variability in cell orientation and aspect ratio for the experimental and simulated morphologies. The values were first calculated separately for each through-thickness bin and subsequently averaged across all bins. The orientation variability is reported as the mean absolute standard deviation in degrees, whereas the aspect-ratio variability is expressed as the mean relative standard deviation. For the experimental results, the values represent the variability among the detected cell profiles from three independently manufactured specimens per condition. For the simulations, the values describe the variability among the resolved bubbles and do not represent numerical uncertainty or variability between repeated simulations.

## 4. Discussion

### 4.1. Evaluation of the Bubble Growth Model and Micro-Scale Simulation

The central finding is that bubble growth can be introduced into the resolved two-phase CFD framework through physically motivated gas mass sources without prescribing an artificial initial overpressure. This supports the working hypothesis that diffusion-driven gas uptake can be coupled to a VoF simulation while retaining the model’s ability to resolve bubble transport, deformation, interaction, and coalescence. Unlike the surrogate growth method used previously, the resulting expansion is continuous and does not generate pronounced pressure or velocity oscillations [27]. The improvement is attributed to the gradual addition of gas mass rather than to an instantaneous pressure imbalance at bubble initialization.

The results further indicate a transition between two growth mechanisms. During the initial growth period, diffusion of dissolved gas into the bubbles provides the dominant contribution to gas-volume increase. As the prescribed bulk concentration approaches equilibrium and the concentration driving force decreases, the calculated mass transfer becomes small. Subsequent expansion is then governed primarily by the decreasing surrounding pressure, while cooling and the associated increase in melt viscosity progressively restrict further growth and deformation. This interaction between diffusion, pressure evolution, and rheological resistance is consistent with the principal mechanisms represented in classical polymer-foam cell models [31,32,33,37]. In contrast to those models, however, the present approach does not require spherical symmetry and therefore permits the simultaneous calculation of deformation and coalescence.

The through-thickness differences in bubble growth demonstrate the importance of resolving the local thermomechanical state. Near the cavity wall, rapid cooling increases the melt viscosity and shortens the available growth period, whereas bubbles in the warmer core remain mobile for longer. This mechanism contributes to the experimentally observed distribution of larger cells in the core and smaller cells toward the compact surface region [7,56,57]. The result indicates that a single set of gas properties can produce spatially varying bubble growth when coupled to the locally resolved temperature, pressure, and flow fields.

Nevertheless, the current initialization procedure limits the predicted cell-size distribution. Only bubbles expected to reach a mesh-resolvable size are introduced, requiring a minimum final size to be selected before the simulation. Sub-grid nuclei and bubbles that stabilize below this threshold are consequently excluded. Moreover, all resolvable bubbles are introduced during one nucleation event with the same initial radius. The relatively homogeneous simulated size distribution within each layer therefore results partly from the initialisation procedure rather than solely from the physical growth process. Secondary and tertiary nucleation would introduce later-forming bubbles with shorter growth times and would likely broaden the predicted cell-size distribution.

The prescribed bulk gas concentration history represents an additional limitation. Although the decreasing concentration reflects consumption of dissolved gas during nucleation and bubble growth, its temporal evolution is currently imposed rather than derived from a species-conservation equation. The model therefore does not resolve local depletion zones, competition for dissolved gas between neighboring bubbles, or the feedback of bubble growth on the remaining bulk concentration. Consequently, the local mass-transfer relation is physically based, but the complete bubble-growth prediction remains conditional on the prescribed bulk gas concentration history. Agreement with the experimental morphology therefore supports the implementation and qualitative behavior of the mass-transfer coupling but does not independently validate the assumed temporal gas-depletion curve. A closed prediction of the gas balance requires the bulk concentration to be calculated from a global gas-mass balance or, preferably, from a transported dissolved-gas-concentration field.

### 4.2. Prediction of Spatial Cell Properties

The combined comparison of cell density, area, aspect ratio, and orientation supports the working hypothesis that characteristic trends in local foam morphology can be derived from the pressure, temperature, and flow histories transferred from the macro-scale process simulation. The micro-scale framework reproduces the principal skin–core characteristics reported for foam-injection-molded parts, including a compact or weakly foamed surface region, smaller cells toward the cavity wall, larger cells in the core, and preferential deformation in the intermediate shear layers [7,56,57]. Importantly, these characteristics are not prescribed as spatial profiles but emerge from the coupled nucleation, mass-transfer, cooling, deformation, and coalescence calculations. The qualitative agreement indicates that the framework represents the principal mechanisms responsible for the development of spatially heterogeneous cell morphology. It does not, however, constitute quantitative validation of the absolute morphology descriptors.

The results also demonstrate that the morphology descriptors must be interpreted jointly. Cell density determines how the available dissolved gas is distributed among the nucleated bubbles and thereby influences the resulting cell size. Cell size, in turn, affects capillary resistance and interfacial area, whereas the local velocity and stress fields govern deformation and orientation. Coalescence couples these quantities further by decreasing cell density while increasing the area and altering the shape of the merged cells. This interaction explains why morphology cannot be described adequately by an isolated prediction of average cell radius or density. The resolved CFD formulation offers an advantage over conventional spherical cell models because it permits the simultaneous evaluation of cell number, growth, shape, orientation, and interaction.

The comparison nevertheless reveals that qualitative agreement in the spatial trends must be distinguished clearly from quantitative predictive capability. The present model does not yet reproduce the absolute cell density, cell area, and aspect ratio consistently across all investigated conditions. Some deviations result from stereological bias because the experimental two-dimensional sections intersect three-dimensional cells whose centers may lie outside the analyzed plane, whereas the simulated bubbles are resolved in their central computational plane. This generally increases the number of observed cell profiles relative to the planar simulation, while off-center intersections reduce their apparent area and may cause them to appear smaller and more circular. In addition, larger cells have a higher probability of being intersected and may therefore be overrepresented in the measured distribution (Figure 8). The apparent aspect ratio and orientation are likewise affected by the three-dimensional cell geometry and the position of the sectioning plane. Furthermore, restricting nucleation and bubble interaction to one computational plane reduces the number of neighboring bubbles and therefore the simulated probability of coalescence. No stereological correction was applied because the deformed, oriented, and coalesced cells do not satisfy the assumptions of a simple spherical stereological model, and their three-dimensional size and orientation distributions are unknown. The comparison is therefore interpreted primarily in terms of relative spatial and process-dependent trends. Quantitative correction and validation will require three-dimensional microscopy or micro-computed-tomography data.

These off-plane intersections of three-dimensional cells and small cells formed by secondary and tertiary nucleation can possibly cause substantially higher experimental cell density obtained for process parameter set A (Figure 8). The higher spatial cell density increases bubble–bubble interactions and restricts bubble expansion in the thickness direction. In particular, the larger core bubbles may restrict the expansion of bubbles in the adjacent regions, thereby increasing the local resistance to growth across the part thickness. Under these conditions, expansion may proceed preferentially in the flow direction, producing stronger alignment even when the deformation is not caused exclusively by shear. The lower in-plane cell density in the simulation provides more space for unconstrained expansion and therefore underrepresents this interaction-induced orientation.

A further contribution may arise from the simulated velocity field. The local flow results from the superposition of the pressure-driven flow imposed through the boundary conditions and the expansion-induced displacement flow generated by bubble growth. The prescribed pressure gradient establishes the net flow direction but may not reproduce the experimental balance between these mechanisms once bubble expansion contributes substantially to melt displacement. Consequently, the simulated shear and elongational-flow profiles in the shear and near-surface regions may differ from those in the experiment, leading to a different cell-orientation distribution. In addition to the dimensional effects, the simplified nucleation history discussed in Section 4.1 influences the quantitative comparison of the morphology descriptors. In particular, the omission of small, late-nucleated cells contributes to the narrower simulated cell-size distribution and may increase the simulated mean aspect ratio relative to the experimental cross-sections. This systematic difference further supports interpreting the comparison in terms of spatial and process-dependent trends rather than absolute morphology values.

The overprediction of the spatial difference between Positions 1 and 2 points to a separate limitation in the macro–micro coupling. The micro-scale simulations respond strongly to the local pressure and temperature histories supplied by the macro-scale model, whereas the experiments show a smaller variation along the flow path. This may indicate that the transferred histories contain spatial differences that are too large, that the micro-scale model is overly sensitive to them, or that both effects contribute. The comparison therefore does not identify a single erroneous material parameter; instead, it shows that quantitative morphology prediction requires consistency between the macro-scale process fields, the micro-scale material response, and the coupling boundary conditions.

The use of different rheological models at the two scales is unlikely to explain the excessive micro-scale velocity through a lower viscosity because the Cross-WLF and PTT models provide comparable shear viscosities under the investigated conditions. Moreover, the additional viscoelastic and extensional stresses represented by the PTT model tend to increase the resistance to bubble deformation rather than reduce it, although a quantitative influence of the different constitutive descriptions cannot be excluded.

The need to reduce the transferred pressure difference reveals a structural limitation of the present one-way coupling. The macro-scale simulation calculates pressure and flow using an averaged bubble-growth model, whereas the micro-scale simulation explicitly resolves the expansion of individual bubbles and the associated displacement of the surrounding melt. During filling and immediately after injection, the flow is predominantly governed by the imposed pressure gradient and residual melt momentum. As foaming progresses, however, bubble expansion becomes an increasingly important source of melt displacement. The micro-scale velocity field then results from both the pressure-driven flow imposed through the boundary conditions and the locally generated expansion-driven flow. Consequently, the unmodified macro-scale pressure difference can lead to excessive melt velocities and bubble deformation.

The previous investigations and the correction applied in the present study confirm that the absolute bubble deformation is highly sensitive to the pressure-driven contribution [27]. The morphology predictions presented here must therefore be interpreted as results obtained under case-specifically corrected local flow conditions rather than as a fully predictive one-way transfer of the macro-scale pressure field. Nevertheless, the comparison with the experiments shows that the corrected simulations reproduce the principal through-thickness trends in cell density, size, orientation, and aspect ratio. The pressure correction therefore enables evaluation of the newly implemented bubble-growth model and its interaction with the local flow field, but it does not validate the present coupling strategy for arbitrary geometries or processing conditions.

A predictive multiscale framework will require feedback from the micro-scale bubble expansion to the macro-scale pressure and volume-flow solution. During the injection-dominated stage, the macro-scale process fields may continue to provide the micro-scale boundary conditions. During the subsequent expansion-dominated stage, however, the gas-volume increase calculated at the micro scale should contribute to the local pressure and flow evolution. This could be achieved through iterative two-way coupling or adaptive boundary conditions that account for the calculated expansion-induced displacement. Such an extension would reduce or eliminate the need for a case-specific pressure correction.

### 4.3. Outlook for Transferring Morphology to the Component Scale

The micro-scale simulations are performed at selected support positions because resolving the complete injection-molded part at the bubble scale would be computationally prohibitive. Nevertheless, the calculated local morphology descriptors, including cell density, cell area, aspect ratio, orientation, and compact-layer thickness, could be transferred to the macro-scale component mesh. Such a transfer was not implemented in the present study but represents a potential application of the developed framework. Ideally, this transfer would be based on similarities in the local process histories rather than on geometric distance alone. Relevant process descriptors could include the temperature at nucleation, pressure-drop rate, cooling time, and integrals of velocity or strain rate after nucleation. This would allow regions with comparable thermomechanical histories to be assigned comparable microstructures, even when they are spatially separated.

If suitable transient process descriptors cannot be exported over the complete macro-scale mesh, the morphology descriptors could initially be interpolated in physical space using the micro-scale simulation positions as support points. Additional support points would be placed in regions with strong process gradients, such as near the gate, flow front, thickness transitions, or rapidly cooled surfaces. The resulting spatial morphology fields could then be used to assign local morphology-dependent mechanical and thermal properties to the part model. This provides a possible route from macro-scale process simulation through local microstructure prediction to microstructure-aware component analysis. However, the interpolation procedure and the resulting property fields still require implementation and validation against spatially resolved experimental measurements.

## 5. Conclusions and Outlook

This study introduces a diffusion-based bubble-growth extension in which bubble-specific mass transfer is calculated from local interfacial conditions using a prescribed time-dependent bulk gas concentration. Diffusion-controlled gas uptake is represented by individual mass sources calculated from the local bubble geometry, temperature, gas pressure, and concentration driving force. In contrast to the previously used initial-overpressure approximation, the new model produces continuous and monotonic bubble expansion without non-physical pressure or velocity oscillations. The results show that the calculated mass transfer constitutes the main contribution to early bubble growth, while the subsequent pressure decrease continues to expand the gas phase after the diffusion-driven mass flow has diminished.

The model qualitatively reproduces essential features of experimentally observed foam morphology. These include larger cells in the core, decreasing cell size toward the cavity surface, process-dependent changes in cell density and area, and preferential cell deformation in the intermediate shear layers. For process parameter set A, the predicted aspect ratios are also comparable in magnitude to the experimental results. These results were obtained using the case-specific pressure-difference correction described in Section 2.4 and therefore demonstrate the capability of the micro-scale model under corrected local flow conditions rather than a fully predictive one-way transfer of the macro-scale pressure field. The comparison nevertheless reveals quantitative deviations in cell density, spatial cell-area variation, and deformation. These deviations are primarily associated with the two-dimensional representation, single-stage nucleation, prescribed bulk gas concentration history, uncertain material parameters, and one-way transfer of macro-scale pressure boundary conditions.

The next development priority should be to close the gas balance by calculating the bulk gas concentration rather than prescribing its temporal development. As an intermediate step, the concentration could be updated using a global gas-mass balance based on the mass transferred into all bubbles. Ultimately, implementing a transported dissolved-gas concentration field directly in OpenFOAM would provide a spatially resolved solution, enforce species conservation, and represent local depletion and competition for gas between neighboring bubbles. The concentration field should also be coupled to melt rheology because dissolved blowing agent can reduce plastic melt viscosity and thereby alter bubble growth and deformation [28,35]. This extension would provide a more consistent coupling between mass transfer, bubble expansion, and flow.

A second priority is to introduce sequential nucleation events. Secondary and tertiary nucleation would broaden the simulated size distribution and include small cells that nucleate later and have less time to grow or deform. Because such cells contribute substantially to experimental cross-sectional statistics, their inclusion is expected to improve the predicted cell density, mean aspect ratio, and coalescence behavior. Extending the model to three dimensions is similarly important for representing spatial cell density, off-plane interactions, realistic coalescence frequencies, and direct comparison with three-dimensional microscopy or micro-computed-tomography data.

Further work should also include a systematic sensitivity analysis of the nucleation and bubble-growth parameters. The diffusion coefficient, solubility, surface tension, contact angle, gas concentration history, relaxation spectrum, and temperature-dependent viscosity should be varied to identify which parameters dominate the predicted morphology. This analysis can guide targeted measurement of material properties and distinguish parameters that require accurate experimental characterization from those that can be estimated without substantially reducing predictive quality. Simulations using alternative viscosity models and parameter sets would additionally clarify the influence of shear thinning, viscoelastic relaxation, and extensional response on bubble deformation.

Finally, the coupling between macro- and micro-scale simulations should be strengthened. A transition criterion should distinguish injection- or inertia-driven flow from the later expansion-driven regime, and an iterative or two-way coupling should allow the micro-scale expansion rate to correct the local macro-scale pressure and flow conditions. After validation for the present low-pressure FIM process, the framework can be extended to other process variants. Particularly, gas-counter-pressure and core-back processes are relevant, where distinct pressure histories and cavity-volume changes generate substantially different nucleation and growth conditions. In its current form, the framework should be regarded as a methodological demonstration that reproduces physically meaningful qualitative morphology trends. With the proposed developments and subsequent quantitative validation, it may provide a basis for spatially resolved prediction of foam morphology and its transfer to mechanical and thermal component analyses.

## Figures and Tables

**Table 1:** Process parameter sets A and B used for experimental part production with the injection molding process and the corresponding macro-scale FIM simulations. The melt temperature, mold temperature, and volumetric flow rate are varied.

Process Parameter	Parameter Set A	Parameter Set B	Unit
Melt temperature	300	280	[°C]
Mold temperature	110	90	[°C]
Volume rate	100	85	[cm^3^/s]
Gas concentration	0.6	0.6	[weight-%]
Switch-over point	95	95	[vol-%]
Holding pressure time	0	0	[s]
Cooling time	25	25	[s]
Mold-opening time	5	5	[s]

**Table 2:** Standard Moldex3D Han and Yoo model parameters used for N_2_ foaming.

Material Parameter	Value	Unit
Gas molecular weight	28	[g/mol]
Gas diffusion coefficient D_0_	1.5 × 10^−6^	[cm^2^/s]
Gas solubility parameter S_0_	2 × 10^−13^	[mol/(cm^3^∙Pa)]
Sigma $\sigma_{ref}$	0.0178	[N/m]
Nucleation correction factor f_0_	7.4 × 10^−25^	[-]
Nucleation correction factor F	0.001	[-]
Nucleation rate threshold J_t_	0.1	[1/(cm^3^∙s)]

**Table 3:** Material parameters of PC, including PTT and Cross-WLF model parameters used for the micro-scale and macro-scale simulations.

Material Parameter	Value	Unit
Thermal conductivity	0.23	[W/(m∙K)]
Heat capacity	2000	[J/(kg∙K)]
PTT $\upsilon_{M}$	0.1	[Pa∙s]
PTT λ	0.004	[s]
PTT ε	0.01	[-]
EOS C_0_	6.257 ∙ 10^−4^	[m^3^/kg]
EOS C_1_	5.837 ∙ 10^−7^	[m^3^/(kg∙K)]
EOS C_2_	−3.327 ∙ 10^−12^	[m^3^/(kg∙K^2^)]
EOS C_3_	−8.115 ∙ 10^−13^	[m^3^/(kg∙Pa)]
EOS C_4_	2.987 ∙ 10^−15^	[m^3^/(kg∙Pa∙K)]
Cross-WLF A_1_	7.9837	[-]
Cross-WLF A_2_	162	[K]
Cross-WLF D_1_	1207	[Pa∙s]
Cross-WLF T_ref_	518	[K]

**Table 4:** Material parameters of N_2_, including temperature-dependent solubility and diffusivity parameters in the plastic melt used for the micro-scale simulation.

Material Parameter	Value	Unit
Prandtl number	0.7	[-]
Specific heat capacity	1045	[J/(kg∙K)]
Molecular weight	28.9	[g/mol]
Reference surface tension $\sigma_{ref}$	0.0178	[N/m]
Reference Temperature for surface tension $T_{\sigma ,ref}$	550	[K]
Temperature coefficient $k_{\sigma }$	0.003	[1/K]
Contact angle [53]	34	[°]
Reference Henry coefficient, S(298 K) [47,54]	9.0	[mol/(m^3^∙MPa)]
Enthalpy of solution, $∆H_{S}$	7000	[J/mol]
Reference diffusivity D_0_ [47]	1.5 ∙ 10^−10^	[m^2^/s]
Reference temperature T_D0_ [47]	473	[K]
Exponent n [47]	23	[-]
Initial bubble radius r_0_	60	[µm]
Viscosity	2.84 ∙ 10^−5^	[Pa∙s]

**Table 5:** Number of bubbles nucleated according to the calculation presented in [27].

Process Parameter Set	Position	Nucleated Bubbles
A	1	65
	2	153
B	1	25
	2	53

**Table 6:** Average variability in cell orientation and aspect ratio across the part thickness. Orientation variability is reported as the absolute standard deviation in degrees, and aspect-ratio variability is reported as the relative standard deviation. The simulated values describe bubble-to-bubble variability.

Type	Parameter Set	Position	Standard Deviation	Relative Standard Deviation
Property	-	-	Orientation [°]	Aspect Ratio [-]
Simulation	A	1	21.7	0.71
	A	2	19.6	0.69
	B	1	25.9	0.65
	B	2	25.5	0.67
Experiment	A	1	21.6	0.39
	A	2	21.3	0.19
	B	1	12.1	0.17
	B	2	13.7	0.31

## Data Availability

The data and materials for this publication are available upon request at the following link: http://hdl.handle.net/21.11102/3bdbcc87-91a4-4530-a3b9-68f487c5a459 (accessed on 7 July 2026).

## Abbreviations

The following abbreviations are used in this manuscript:

CFD	Computational fluid dynamics
DFG	Deutsche Forschungsgemeinschaft
EOS	Equation of state
FEM	Finite element method
FIM	Foam injection molding
N_2_	Nitrogen
PC	Polycarbonate
PTT	Phan–Thien–Tanner
VoF	Volume of fluid
VTK	Visualization toolkit
